# High-Performance Wrap-Gated InGaAs Nanowire Field-Effect Transistors with Sputtered Dielectrics

**DOI:** 10.1038/srep16871

**Published:** 2015-11-26

**Authors:** Li-Fan Shen, SenPo Yip, Zai-xing Yang, Ming Fang, TakFu Hung, Edwin Y.B. Pun, Johnny C. Ho

**Affiliations:** 1 Department of Electronic Engineering, City University of Hong Kong, 83 Tat Chee Avenue, Kowloon, Hong Kong; 2 Department of Physics and Materials Science, City University of Hong Kong, 83 Tat Chee Avenue, Kowloon, Hong Kong; 3 State Key Laboratory of Millimeter Waves, City University of Hong Kong, 83 Tat Chee Avenue, Kowloon, Hong Kong; 4 Shenzhen Research Institute, City University of Hong Kong, Shenzhen, China

## Abstract

Although wrap-gated nanowire field-effect-transistors (NWFETs) have been explored as an ideal electronic device geometry for low-power and high-frequency applications, further performance enhancement and practical implementation are still suffering from electron scattering on nanowire surface/interface traps between the nanowire channel and gate dielectric as well as the complicated device fabrication scheme. Here, we report the development of high-performance wrap-gated InGaAs NWFETs using conventional sputtered Al_2_O_3_ layers as gate dielectrics, instead of the typically employed atomic layer deposited counterparts. Importantly, the surface chemical passivation of NW channels performed right before the dielectric deposition is found to significantly alleviate plasma induced defect traps on the NW channel. Utilizing this passivation, the wrap-gated device exhibits superior electrical performances: a high *I*_ON_/*I*_OFF_ ratio of ~2 × 10^6^, an extremely low sub-threshold slope of 80 mV/decade and a peak field-effect electron mobility of ~1600 cm^2^/(Vs) at *V*_DS_ = 0.1 V at room temperature, in which these values are even better than the ones of state-of-the-art NWFETs reported so far. By combining sputtering and pre-deposition chemical passivation to achieve high-quality gate dielectrics for wrap-gated NWFETs, the superior gate coupling and electrical performances have been achieved, confirming the effectiveness of our hybrid approach for future advanced electronic devices.

In the past decade, III–V compound semiconductor nanowires (NWs) have attracted extensive amount of research and development interest due to their excellent physical properties for high-performance nanoelectronics and highly efficient photovoltaics^1^,^2^,^3^,^4^,^5^,^6^,^7^,^8^,^9^,^10^. Among many III–V NW materials, InAs has been demonstrated with the superior field-effect electron mobility (*μ*) as well as the gigahertz device operation^11^,^12^,^13^. However, further applications are still restricted by the substantial leakage current in InAs-based devices because of its small electronic band gap^14^,^15^. Recently, ternary InGaAs NWs with uniformly tunable chemical stoichiometries have been successfully illustrated as the alternative device channel material, and the relatively larger band gap reduces the leakage issue but not the electron mobility^16^,^17^,^18^,^19^,^20^,^21^. At the same time, a newly developed gate-all-around (GAA) device architecture with a coaxial wrap gate completely surrounding the NW channel is also frequently adopted. The capacitive coupling between the gate and the NW is increased in order to reduce the OFF current (*I*_OFF_) and improve the sub-threshold slope (*SS*) through the enhanced electrostatic gate control over the channel^22^,^23^,^24^,^25^. It is noted that this wrap-gated (WG) device geometry depends on the conformal deposition of high-quality and ultrathin (i.e. ~10 nm) high-k dielectric layer all over the NW, in which the resulting device characteristics are strongly affected by the obtained dielectric/NW interface qualities; as a result, atomic layer deposition (ALD) is typically employed for this critical dielectric coating step despite its high processing cost, long reaction time, and limited material choices for the dielectrics^26^.

Both vertical and lateral WG NW device configurations have been reported for InAs^27^,^28^,^29^,^30^,^31^,^32^,^33^. Wernersson *et al.* provided a comprehensive investigation of vertical WG InAs NW field-effect transistors (FETs) with the HfO_2_ gate dielectric, delivering a maximum ON current density of 0.08 A/mm with an *SS* of 75 mV/dec at room temperature^34^. This *SS* value is very close to the theoretical limit of *SS *= 60 mV/dec, indicating good control of channel electrostatics and interface properties. However, electron beam lithography was required to pattern growth seeds at specified locations for the vertical NW synthesis on underlying crystalline III–V substrates^27^, together with complicated device fabrication process with numerous lithography and etching steps^28^, all these made the implementation of these vertical device structures challenging. On the other hand, Deshmukh and his coworkers attained lateral InAs WG NWFETs with only two lithography steps and without any etching requirement, the devices exhibit a respectable ON-OFF current ratio (*I*_*ON*_/*I*_*OFF*_) of 5 × 10^3^ at *V*_*DS*_ = 0.5 V at room temperature and an impressive *SS* of 5–54 mV/dec at low temperatures (1.5 to 250 K)^30^. During the device fabrication, it is crucial to use a low-temperature process (i.e. <120 ^o^C) for the HfO_2_ deposition to avoid polymerizing the resist layers employed in the lithography steps. Similarly, Xu *et al.* further optimized the device performance, yielding a significantly large current density of 400 μA/μm, an impressive *I*_*ON*_/*I*_*OFF*_ of ~10^4^ and a *μ* of 1600 cm^2^/(Vs) which is the highest among all WG InAs NWFETs reported so far for *V*_*DS*_ = 0.5 V at room temperature^33^. Up to now, there have been few reports focusing on using the improved channel materials of InGaAs NWs with further simplified WG device fabrication process.

Recently, we have successfully synthesized crystalline InGaAs NWs, with a smooth surface and a low defect concentration, using a unique two-step catalytic chemical vapor deposition (CVD)^18^. In this work, we use the crystalline NWs to construct lateral WG NWFETs with a simple fabrication process. Notably, the high-κ gate dielectric (~12 nm thick Al_2_O_3_) can be conformally deposited around the suspended NW channel by standard sputtering, which is a simple, attractive and economic process to deposit dielectrics in industry. Combining with the pre-deposition surface passivation of NWs by self-assembly sulfur-containing monolayer such as ammonium sulfide ((NH_4_)_2_S), the fabricated devices are found to display improved dielectric/NW interface properties, exhibiting excellent electrical performances such as a small *I*_OFF_ below 1 pA, a high *I*_ON_/*I*_OFF_ ratio of ~2 × 10^6^, a low *SS* of 80 mV/dec, and an excellent *μ* approaching 1600 cm^2^/(Vs) at *V*_*DS*_ = 0.1 V at room temperature. This superior capacitive gate coupling of WG InGaAs NWFETs confirms the versatility of our simplified WG device fabrication scheme using high-quality sputtered dielectrics, as well as the potential applications of InGaAs NW channels for future high-speed, low-power, and high-frequency electronic devices.

## Results

**Figure 1:**
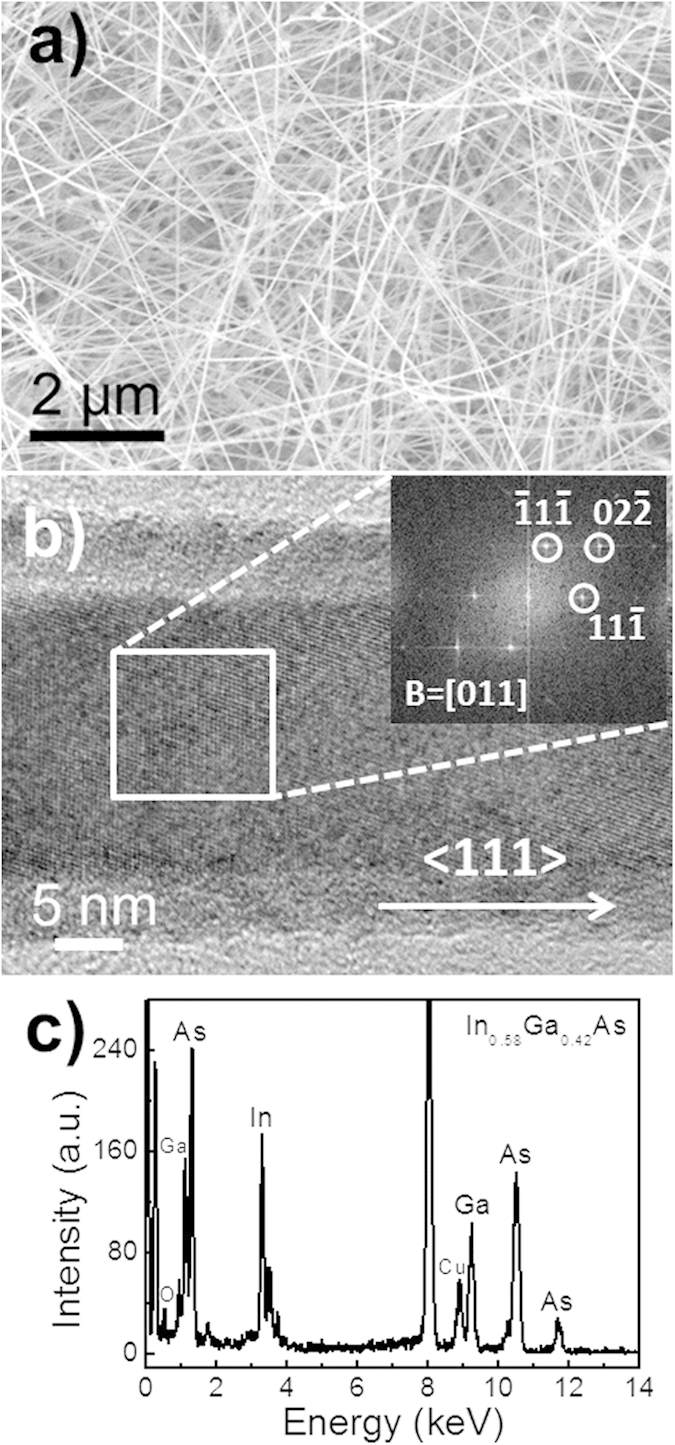
(**a**) Electron microscopy characterization of the as-grown InGaAs NWs; (**b**) High-resolution transmission electron microscope (HRTEM) image and the corresponding fast Fourier transform (FFT) of a representative NW; (**c**) EDS spectrum of the corresponding NW body.

The InGaAs NWs, synthesized by the two-step catalytic CVD method in this work, are dense, long (>10 μm), and straight with the smooth surface, as shown in the scanning electron microscope (SEM) image in Fig. 1a. In order to evaluate the crystallinity of the as-grown NWs, high-resolution transmission electron microscopy (HRTEM) are performed on a representative NW, as illustrated in Fig. 1b. Based on the plane spacing determination and the reciprocal lattice spots extracted by fast Fourier transform (FFT), the NW exhibits single-crystalline zinc-blende (ZB) structure with a dominant growth orientation in the <111> direction, and no significant amount of stacking faults or twin-plane polytypic defects are found in the samples. The spacing between the adjacent lattice planes are found to be 0.34 and 0.21 nm, which are in good agreement with the plane spacing of (111) and (022) equivalent planes in the In-rich thin-film counterparts, respectively^35^. Also, the NW stoichiometry can be assessed using the corresponding energy-dispersive X-ray spectroscopic (EDS) spectrum depicted in Fig. 1c. The In concentration *x*, being 0.58 in our ternary In_*x*_Ga_1−*x*_As NWs, is consistent with our previous work in controlling the NW composition by varying the precursor source powder ratio^17^.

**Figure 2:**
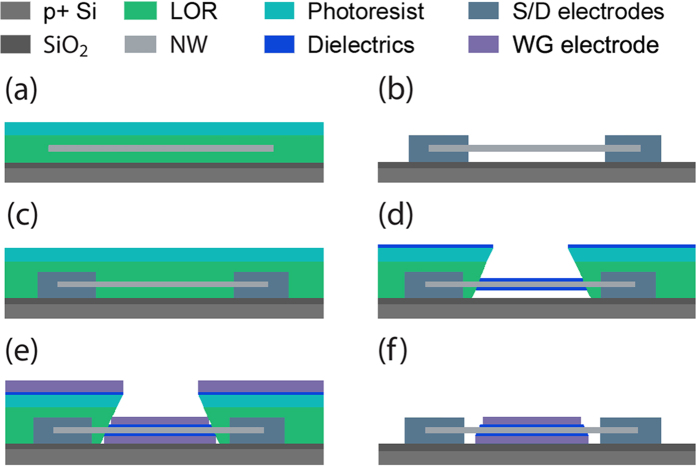
Schematic diagrams for the fabrication process of InGaAs wrap-gated NWFET devices. (**a**) Sandwiched NW between lift-off resists covered by a layer of photoresist; (**b**) S/D electrodes defined by lithography and processed with the (NH_4_)_2_S passivation; (**c**) Spin-coated with LOR and photoresist; (**d**) Window opened by lithography and Al_2_O_3_ thin film deposited homogeneously by sputtering; (**e**) Deposition of Ti/Al gate metal by sputtering; (**f**) Final lift-off.

Next, the WG InGaAs NWFETs can be fabricated by a simple process as presented in Fig. 2. Briefly, the as-prepared NWs are first drop-casted onto the SiO_2_/p^+^-Si substrate (50 nm thick thermally grown oxide) pre-coated with a layer of 100 nm thick lift-off resist (LOR). After the NW deposition, another stack of LOR / photoresist (100 nm / 500 nm thick) layer is spin-coated to form a sandwiched structure (Fig. 2a). Standard photolithography is then employed to define the source/drain (S/D) electrodes, followed by Ni evaporation (150 nm thick) and lift-off process, with the purpose to achieve a suspended NW structure (Fig. 2b). Here, this suspended structure is soaked in (NH_4_)_2_S (stock solution) for 40 s, carefully rinsed in DI water and anhydrous ethanol, and baked at 120 ^o^C for 5 hours in order to passivate the exposed NW surface. A second photolithography step is then performed to outline the WG region, and a ~12 nm thick Al_2_O_3_ high-k dielectric layer is deposited around the NW by conventional sputtering with a slow deposition rate of ~0.5 nm/min (Fig. 2d). Finally, gate stack electrode (5 nm Ti / 90 nm Al) is sputtered with a slow deposition rate of ~2 nm/min at the beginning to ensure conformal contact with the Al_2_O_3_ layer and realize the final WG geometry (Fig. 2e,f). The substrate rotation is crucial throughout the deposition process in order to ensure conformal coverage of Al_2_O_3_ and gate stack onto the NW channel.

**Figure 3:**
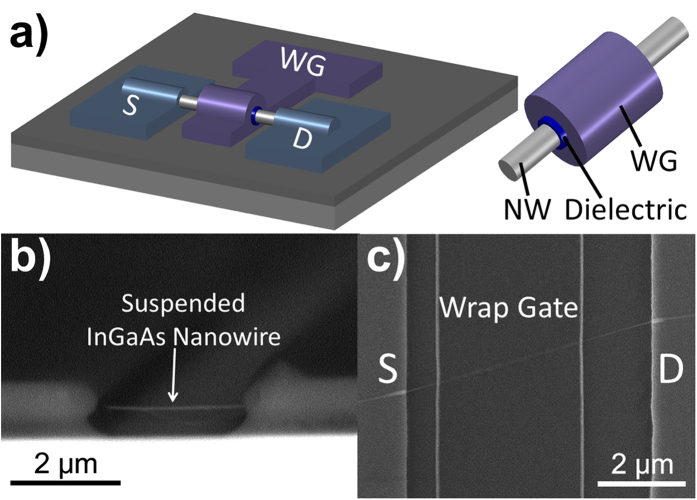
(**a**) Illustrative schematic of the InGaAs wrap-gated NWFET device; (**b**) SEM image of a suspended InGaAs NW channel; (**c**) SEM image of a representative InGaAs wrap-gated NWFET fabricated.

Figure 3a shows the illustrative schematic of the WG InGaAs NWFET constructed in this work, and Fig. 3b,c give the cross-sectional and top-view SEM images of a representative device before and after the WG fabrication, respectively. It is noted that the NW device has a typical diameter of *d* ~29 nm and a WG length of *L* ≈ 3.67 μm (distance between source/drain ~6 μm). Although high-quality gate dielectrics can be often obtained by the ALD method, the ALD processing is complicated involving a lot of organometallic precursors and requiring a lot of calibrations before achieving the optimal condition. Importantly, the precursor residues may also be incorporated in the deposited films. Instead, magnetron sputtering is employed in this work to deposit the dielectric layer around the suspended NW channel, and the process is simpler, more economical, and industrial friendly than the ALD process. Moreover, sputtering is also more compatible with temperature sensitive flexible substrates^36^, enabling the fabrication of mechanically flexible high-performance WG devices. With the aim to inspect the electrical property of the sputtered Al_2_O_3_ dielectric obtained here, simple metal-oxide-semiconductor (MOS) capacitors are fabricated on highly doped p-Si (100) substrate with a resistivity of 0.001 to 0.005 ohm-cm utilizing the same sputtered Al_2_O_3_ dielectric and Al metal electrode. Based on the high-frequency (1 MHz) capacitance−voltage (*C*−*V*) measurement (Supplementary Information Figure S1a), the dielectric constant of sputtered Al_2_O_3_ is determined to be 7.216, which is similar to the value attained by typical ALD process, confirming the excellent dielectric quality of the sputtered Al_2_O_3_ layer used in this work. Furthermore, this dielectric layer also increases the ON current density and minimizes the hysteresis effect of the fabricated device by isolating the NW channel from the external environment, and improves the overall device performance and reliability (Supplementary Information Figure S1b)^3^.

**Figure 4:**
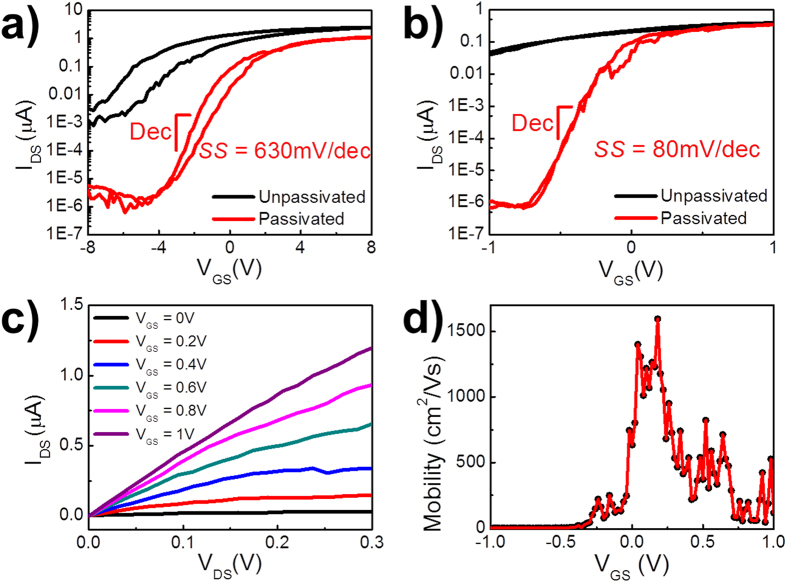
(**a**) Transfer characteristics of the back-gated InGaAs NWFET before and after passivation in logarithmic scale at *V*_DS_ = 0.1 V; (**b**) Transfer characteristics of the InGaAs wrap-gated NWFET with and without passivation in logarithmic scale at *V*_DS_ =0.1 V. (**c**) Output characteristics of the InGaAs wrap-gated NWFET device processed with passivation; (**d**) Mobility assessment of InGaAs wrap-gated NWFET device processed with passivation under *V*_DS_ = 0.1 V at room temperature.

The plasma generated during the sputter deposition of Al_2_O_3_ can damage the NW channel surface by inducing significant amount of surface/interface defect trap states^37^. In order to reduce this negative impact, the surface passivation of III–V NWs by sulfur based chemicals such as ammonium sulfide ((NH_4_)_2_S) have been commonly adopted in other applications, in order to enhance the device performance by improving the *SS* and increasing the *μ*^38^,^39^,^40^,^41^,^42^. Thus, in this work, we employ similar chemical passivation technique with the intention to alleviate the detrimental effect of the plasma induced surface defects here. In details, as shown in Figure 4a, the influence of (NH_4_)_2_S passivation on the InGaAs NW electrical properties is investigated, and the electrical performance of back-gated NWFET before (unpassivated) and after passivation (passivated) is evaluated. It can be clearly seen that after passivation the *I*_OFF_ is reduced significantly and the ON current (*I*_ON_) does not change much. Hence, surface passivation does enhance the device *I*_ON_/*I*_OFF_ by at least two orders of magnitude and decrease the *SS* by half, which can be attributed to the saturation of surface states on the NW channel surface^43^. Accordingly, the device hysteresis can also be reduced by isolating the NW surface from the ambient environment^44^.

As presented in Fig. 4b, when the passivation scheme is applied to the WG device fabrication, the performance enhancement is even more profound due to the larger dielectric/channel interfacial area and better electrostatic gate control. After passivation, the *I*_OFF_ is decreased drastically and the electrical properties such as *SS* and *I*_ON_/*I*_OFF_ are all improved, indicating the significance of surface passivation for the WG device geometry. The device exhibits n-type conduction with a minimal hysteresis under low-voltage operation (*V*_DS_ = 0.1 V), and an extremely small OFF current (*I*_OFF_ = 0.4 pA) with *V*_GS_ = –1 V, and ~1 μA ON current with *V*_GS_ = 1 V with a high *I*_ON_/*I*_OFF_ ratio of ~2 × 10^6^ at room temperature are obtained. Different back-gate bias with *V*_BG_ = 0, 2, 4 V are also applied to the WG device (see Supplementary Information Figure S2), and a negligible influence of *V*_BG_ on the electrical performance of WG devices is observed, indicating the dominant gate control of the WG here. Figure 4c presents the linear *I*_DS_–*V*_DS_ behavior of the same WG device under *V*_DS_ = 0.1 V after passivation, which confirms the ohmic-like contact formation with Ni S/D electrodes and suggests that the passivation process has no adverse effect on the contact properties of the devices.

As an important parameter for low-power and high-speed operations, *SS* characterizes how fast a FET can be switched, and is defined as *SS* = *dV*_GS_/*d*log*I*_DS_, and the steep sub-threshold slope indicates a faster transition between the device OFF and ON states^45^. Although sputtered Al_2_O_3_ dielectric is employed in this work, the fabricated InGaAs WG NWFET exhibits a good *SS* of 80 mV/dec with *V*_DS_ = 0.1 V at room-temperature approaching the theoretical limit of 60 mV/decade, and is even smaller than the recently reported values of ALD enabled InAs WG NWFETs^32^,^33^,^46^, as shown in Fig. 4b. It is also noted that many of our fabricated devices display *SS* values below 200 mV/dec with *V*_DS_ = 0.1 V, illustrating the effectiveness of our combined sputter deposition and chemical passivation approach in achieving high-quality dielectric layers (Supplementary Information Figure S3).

The corresponding field-effect electron mobility of the InGaAs WG NWFET can also be assessed by the standard square law model as demonstrated in Fig. 4d:


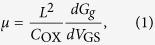


where *L* is the length of the gate, *G*_*g*_ is the conductance of the nanowire channel covered by the gate and *C*_OX_ is the gate capacitance obtained from a finite element analysis software COMSOL tailored for this wrap-gated geometry with the details given in the Supplementary Information Figure S4 and S5. The *C*_OX_ determined from the simulation gives a relatively accurate gate capacitance, together with the known gate length and diameter, the field-effect mobility of the NW can be calculated reliably^12^. It should be noted that there is always a thin native oxide layer of ~2.5 nm on the NW surface^18^, which would be subtracted from the measured NW diameters to give the effective channel width for subsequent mobility determination. As the *G*_*g*_ is only attributed to the conductance of gate coverage area and the two ungated segments in the channel are not controlled by the gate, the conductance of these two ungated parts (*G*_*1*_ and *G*_*2*_) which are independent of *V*_GS_ can be deduced from the total conductance of the nanowire (*G*_*t*_). We assume that when the current nearly saturates, flat band condition occurs and the NW conducts homogeneously in the entire channel. The conductance of the two ungated parts can then be calculated by using the following equation:


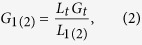


where *L*_1(2)_ is the length of the ungated segment, and *L*_*t*_ is the total length of the nanowire in the channel. The total conductance (*G*_*t*_) can be expressed by


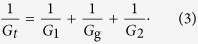


With the conductance of the two ungated parts *G*_*1*_ and *G*_*2*_, the conductance *G*_*g*_ can be calculated by the following equation:


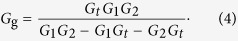


The peak transconductance 
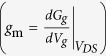
 for the representative WG NWFET device (Figure 4b,d) is found to be 2.52 μS and the calculated peak *μ* is ~1600 cm^2^/(Vs) at room temperature, which is comparable to the lately reported state-of-the-art InAs WG NWFET^33^.

To evaluate the performance enhancement between the back-gated (without any surface passivation) and WG NWFETs, several device parameters such as *I*_ON_/*I*_OFF_, sub-threshold slope, peak transconductance, and peak field-effect electron mobility of the devices are compiled and compared in Table 1 (see details in Supplementary Information Table S1 and Table S2). The average *I*_ON_/*I*_OFF_ of WG devices is found to be ~3.4 × 10^5^, which is an order of magnitude higher than the value of back-gated devices (~5.4 × 10^4^). The *SS* of WG devices varies from 80 to 300 mV/dec and the average values is estimated to be ~200 mV/dec, which is much smaller than that of ~1000 mV/dec for back-gated devices. Moreover, the average peak transconductance of the WG devices is also significantly higher than that of other back-gated devices, and all these improved device performances indicate the excellent capacitive gate coupling of the WG NWFETs fabricated in this work.

## Discussion

Although WG NWFETs have been explored as an ideal FET geometry for low-power and high-frequency applications, the required performance improvement and practical device implementation still lag behind expectation, largely due to the electron scattering at the NW surface and/or interface traps between the NW channel and gate dielectric, and the complicated device fabrication scheme. The current work adopts a hybrid approach, combining the well-established sputter deposition to achieve high-quality Al_2_O_3_ dielectric layers and the pre-deposition surface passivation of NW channels by self-assembly sulfur-containing monolayer in the device fabrication of InGaAs WG NWFETs. The devices are all fabricated using single-crystalline ternary InGaAs NWs with tunable chemical stoichiometry, smooth surface and low defect concentration using a simple process with standard photolithography and electrode deposition. The high-k gate dielectric Al_2_O_3_ layer is deposited homogeneously around the NW channel by conventional sputtering, which is a more economic industrial friendly process as compared to the typical ALD scheme in obtaining the dielectrics. The pre-sputtering chemical passivation by (NH_4_)_2_S is also employed to alleviate the detrimental effect of the plasma induced surface/interface defect trap states on the NW channels. After passivation, the InGaAs WG NWFETs exhibits excellent electrical properties, with a high *I*_ON_/*I*_OFF_ ratio of ~2 × 10^6^, a low *SS* of 80 mV/decade, a small *I*_OFF_ of 0.4 pA, and a peak field-effect mobility of ~1600 cm^2^/(Vs) at *V*_DS_ = 0.1 V at room temperature, which is comparable to or even better than state-of-the-art ALD enabled WG NWFETs^30^,^32^,^33^. Further improvement could be achieved by employing the aromatic thiolate (ArS^−^) based molecular monolayers as the surface modification of NWs in this wrap-gated device fabrication, since this ArS^−^ surface processing can not only decrease the amount of surface traps for the better mobility but also induce the surface electronic charge to move the device *V*_Th_ positively^38^. The superior capacitive gate coupling of WG NWFETs and the improved electrical performance, including the low leakage current and steep sub-threshold slope, suggest the technological potential of our sputtering enabled WG NWFETs for future high-speed, low-power, and high-frequency electronic devices.

## Method

### Nanowire Synthesis

InGaAs nanowires (NWs) used in this study were synthesized in a two-zone horizontal tube furnace by using a solid-source chemical vapor transport method as previously reported^18^. Briefly, the SiO_2_/Si growth substrate (50 nm thermal oxide on degenerately boron doped Si (100) with a resistivity of 0.001 to 0.005 ohm-cm) was pre-deposited with a 0.5 nm thick Au film as the catalyst in thermal evaporator, and then positioned in the downstream zone of the furnace. InAs (99.999% purity) and GaAs (99.999% purity) powders were mixed in 1:1 weight ratio and loaded into a boron nitride crucible in the upstream zone. Hydrogen (99.9995%) was used as carrier gas to transport the evaporated source materials to the growth substrate. A two-step growth method was adopted to ensure the uniform NW morphology and stoichiometry^17^. During the growth, the flow rate of H_2_ was maintained at 100 sccm and the corresponding pressure downstream is ~1 Torr. After the growth, the source and substrate heater were stopped together and the grown NWs were taken out of the furnace after the system was cooled naturally to room temperature under the hydrogen flow.

### Material Characterizations

All material characterizations were performed on the NWs obtained in the 0−1 cm region of growth substrates in order to establish a consistent study. Surface morphologies of the grown NWs were examined with a scanning electron microscope (SEM, FEI/Philips XL30 ESEM-FEG). HRTEM image was observed with a JEOL 2100F transmission electron microscope. The composition of NWs was determined using an energy dispersive X-ray spectroscopy (EDS) detector attached to the JEOL 2100F to measure the chemical composition of the grown NWs. In the EDS measurements, around thirty NWs were randomly chosen as candidates for the EDS point scan in the NW body.

### Nanowire FET Fabrication and Measurements

After CVD growth, the InGaAs NWs were first harvested by sonication in high-purity ethanol solution, and then the InGaAs NW field-effect-transistors (FETs) were fabricated based on both back-gated and wrap-gated FET configuration. For the fabrication of back-gated NWFETs, the obtained NW suspension was randomly drop-casted onto pre-cleaned highly boron doped Si (100) with a resistivity of 0.001 to 0.005 ohm-cm with a 50 nm thick thermally grown gate oxide, and then spin-coated with LOR and AZ5206 photoresist and exposed to ultraviolet light and went through developing. After we delineated the source and drain patterns, a 50 nm thick Ni film was thermally deposited as the contact electrodes followed by a lift-off process.

For wrap-gated NWFETs, the InGaAs NWs were drop-casted onto the SiO_2_/Si substrates which were pre-spin coated by a layer of 100 nm thick LOR, and then spin-coated with LOR and AZ5206 photoresist respectively to form a sandwiched NW between lift off resists, as schematically shown in Fig. 2(a). Photolithography was then utilized to define the source and drain regions, and a 150-nm thick Ni film was thermally deposited as the contact electrodes followed by a lift-off process. Figure 2(b) illustrates the NW is suspended between the electrodes in the developed region, and this suspended configuration was confirmed by the SEM image of a suspended InGaAs NW in Fig. 3(b). Then the devices were soaked in (NH_4_)_2_S solution (stock solution) for 40 s in order to passivate the surface of NWs. After passivation, the devices were rinsed carefully using DI water and ethanol and baked at 120 °C for ~5 h. A second photolithography was performed to define the wrap-gate regions, and a dielectric layer of ~12 nm thick Al_2_O_3_ was deposited homogeneously around the NW by DC sputtering with Al metal target in oxygen and argon ambient (O_2_ to Ar ratio 1.5:25) and then the devices were baked at 100 ^o^C for ~5 h, as presented in Fig. 2(c,d). After that, 5 nm Ti and 90 nm Al were deposited isotropically in a DC sputtering system as the gate electrodes followed by a lift-off process (Fig. 2(e,f)). Electrical performance of the fabricated NWFET devices was characterized with a standard electrical probe station and an Agilent 4155C semiconductor analyzer at room temperature. High-frequency (HF) capacitance-voltage (C–V) measurements were performed using HP4284A precision LCR meter.

## Additional Information

**How to cite this article**: Shen, L.-F. *et al.* High-Performance Wrap-Gated InGaAs Nanowire Field-Effect Transistors with Sputtered Dielectrics. *Sci. Rep.* **5**, 16871; doi: 10.1038/srep16871 (2015).

## Supplementary Material

Supplementary Information

## Figures and Tables

**Table 1:** Compilation and comparison of average on-off ratio, sub-threshold slope (*SS*), peak transconductance and peak field-effect electron mobility between back-gated (without any surface passivation) and wrap-gated devices.

	*I*_ON_/*I*_OFF_	*SS* .(mV/dec)	Peak Transconductance (μS)	Peak Mobility (cm^2^/(Vs))
Back-gated devices	54000	1000	0.18	1050
Wrap-gated devices	340000	200	1.59	1300
