# Supplementary material for: High-Performance Wrap-Gated InGaAs Nanowire Field-Effect Transistors with Sputtered Dielectrics
Source: Sci Rep. 2015 Nov 26;5:16871. doi: 10.1038/srep16871 (PMC4660349; doi:10.1038/srep16871)
Supplement: Supplementary Information [file srep16871-s1.pdf]

High-Performance Wrap-Gated InGaAs Nanowire Field-Effect Transistors with Sputtered Dielectrics

Li-Fan Shen[1,3], SenPo Yip[2,3,4], Zai-xing Yang[2,3,4], Ming Fang[2,4], TakFu Hung[2],

Edwin Y.B. Pun[1,3,*], and Johnny C. Ho[2,3,4,*]

[1] *Department of Electronic Engineering, City University of Hong Kong, 83 Tat Chee Avenue, Kowloon, Hong Kong.*

[2] *Department of Physics and Materials Science, City University of Hong Kong, 83 Tat Chee Avenue, Kowloon, Hong Kong.*

[3] *State Key Laboratory of Millimeter Waves, City University of Hong Kong, 83 Tat Chee Avenue, Kowloon, Hong Kong.*

[4] *Shenzhen Research Institute, City University of Hong Kong, Shenzhen, China.*

SUPPLEMENTARY INFORMATION

* Correspondence and requests for materials should be addressed to J.C.H. (email: johnnyho@cityu.edu.hk) and E.Y.B.P (email: eeeybpun@cityu.edu.hk).

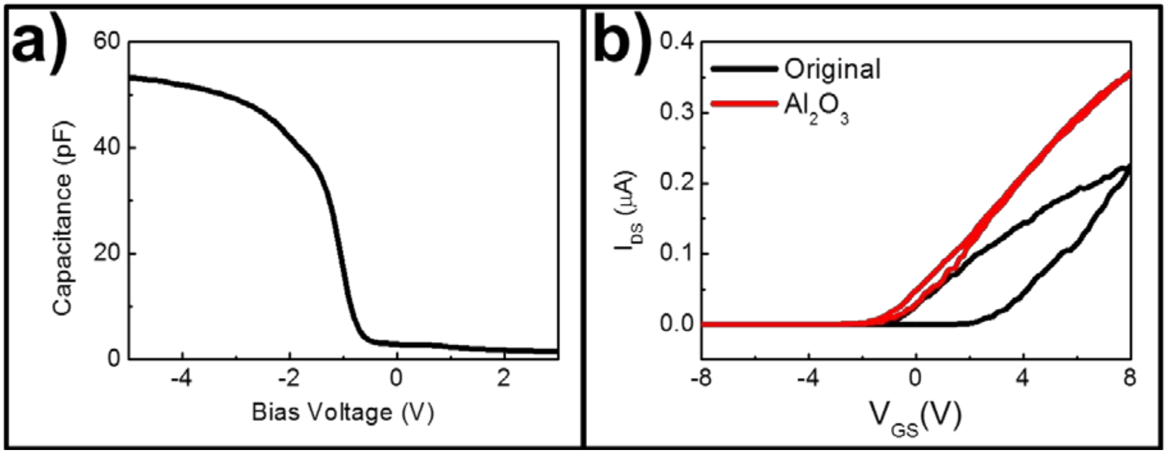

Figure S1. (a) Capacitance-Voltage curve of metal-oxide-semiconductor (MOS) capacitors utilizing the 12nm thick Al_2O_3 deposited by sputtering measured at 1MHz; (b) I_{DS}-V_{GS} curves of InGaAs NWFET before and after the Al_2O_3 deposition by sputtering (V_{DS} =0.1 V).

In order to investigate the electrical property of the Al_2O_3 deposited by sputtering as the dielectrics for InGaAs wrap-gated NWFET devices studied in this work, the simple metal-oxide-semiconductor (MOS) capacitors are fabricated on the p-type (100) bare Si wafer. The 12nm thick Al_2O_3 layer is deposited on the surface of wafer in oxygen and argon ambient by sputtering. After baking 5h at 100°C, Al is deposited on the top of Al_2O_3 by sputtering and patterned as the gate electrode with an area of $10^{-8}\,m^2$, and a low-resistance ohmic contact is formed by sputtering Al on the backside of the wafer. In the high-frequency (1MHz) capacitance–voltage measurement (Figure S1a), the variation in capacitance versus voltage is caused by changes in the space charge region of the moderately doped p-Si (100) semiconductor substrate. At large positive potentials where the silicon surface is in accumulation and there is no space charge region, the capacitance measured is the capacitance of the Al_2O_3 film. The dielectric constant can then be calculated as 7.216 by using $k = C_{OX} d / \varepsilon_0 A$, where C_{OX} is capacitance, d is thickness of the film, ε_0 is the permittivity of free space, and A is the area of the capacitor.

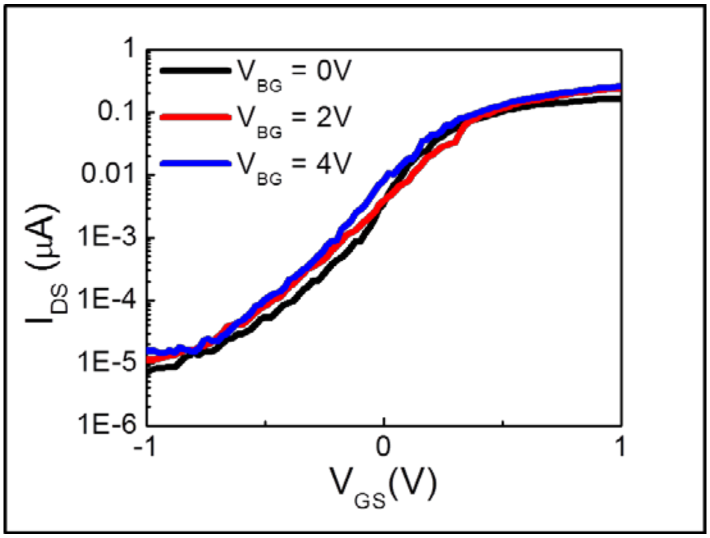

Figure S2. I_{DS}–V_{GS} curves of the InGaAs wrap-gated NWFET device with different back-gate bias at V_{DS} =0.1 V

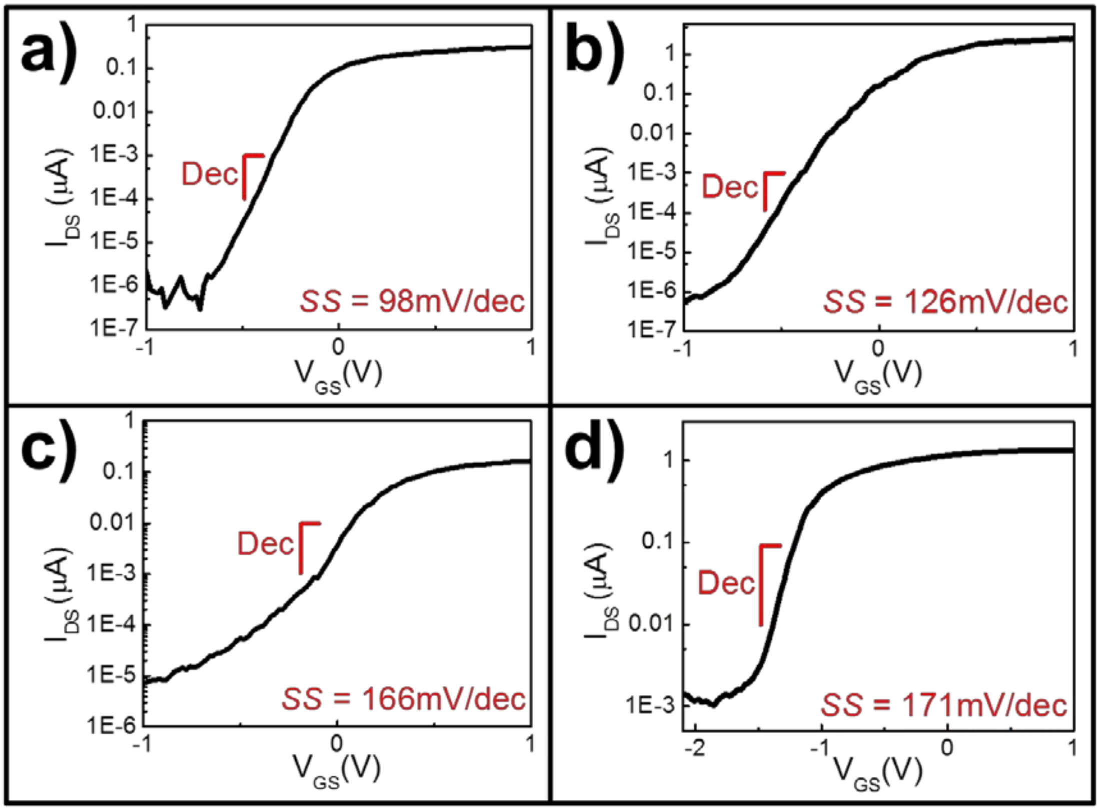

Figure S3. Typical I_{DS}–V_{GS} curves of the InGaAs wrap-gated NWFETs with different sub-threshold slopes at V_{DS} = 0.1 V.

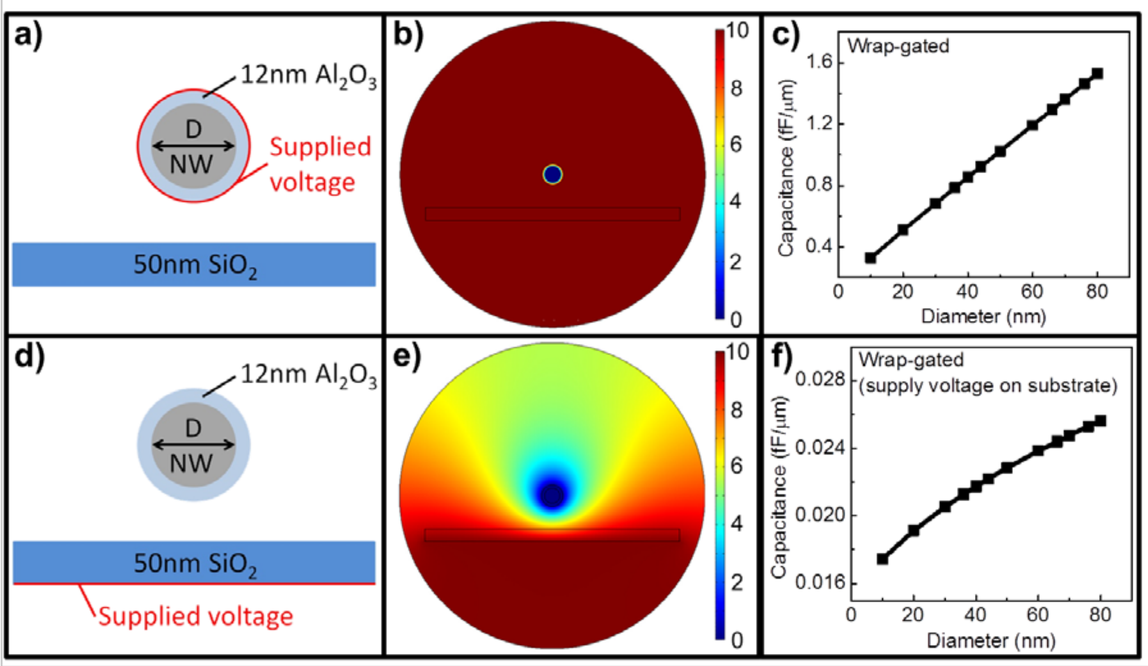

Figure S4. (a, d) Cross-sectional schematic of the NW channel in wrap-gated NWFETs with supplied voltage on the wrap gate and substrate, respectively (not in scale; with the NW diameter of 60nm, SiO_2 thickness of 50nm, ε_{SiO2}~ 3.9, dielectric thickness of 12nm Al_2O_3, ε_{Al2O3}~ 7.216 obtained from the C-V measurement). (b, e) 2D electric potential contour of the corresponding NWFET channel shown in panel a and d, simulated by COMSOL software with the channel length of 10 µm. The corresponding simulated gate oxide capacitance (C_{ox}) per unit of channel length is ~ 1.19 fF/µm for (a) and ~0.02 fF/µm for (d). (c, f) Simulated C_{ox} as a function of diameter per unit of the channel length for both structures, accordingly.

COMSOL Multiphysics is utilized to simulate the gate oxide capacitance (C_{ox}). The simulation is carried out on the wrap-gated NWFET structure with supplied voltage on wrap gate and on substrate as shown in Figure S4 (a, d). Specifically, the NW charge density is assumed to be very high such that the semiconducting NW can be treated as metallic and the NW was modeled as a perfect cylinder. The element size is set to the following values: maximum element size of 804 nm, minimum element size of 3.6 nm, maximum element grow rate: 1.3, resolution of curve of 0.3, resolution of narrow region of 1. The dielectric constant of Al_2O_3 is calculated to be 7.216 by the capacitance–voltage curve mentioned before. The C_{ox} can then be obtained from the simulated electrical field at the voltage of 1V when the NW is set to the ground. Since the C_{ox} of wrap-gated NWFET with supplied voltage on the gate is found much larger than the wrap-gated NWFET with supplied voltage on the substrate, the contribution of C_{ox} from the back-gate can be ignored in the wrap gate device calculation.

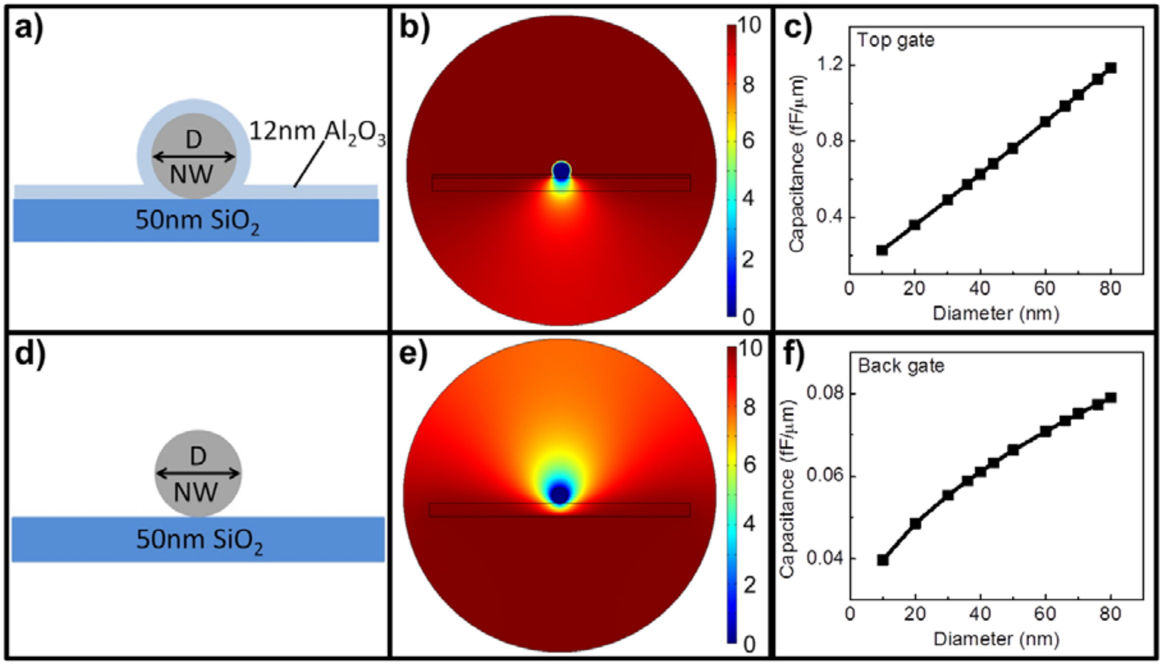

Figure S5. (a, d) Cross-sectional schematic of the NW channel in top-gated and back-gated NWFETs, respectively (not in scale; with the NW diameter of 60nm, SiO_2 thickness of 50nm, ε_{SiO2}~ 3.9, dielectric thickness of 12nm Al_2O_3, ε_{Al2O3}~ 7.2). (b, e) 2D electric potential contour of the corresponding NWFET channel shown in panel a and d at the zero gate bias, simulated by COMSOL software with the channel length of 10 µm. The corresponding simulated gate oxide capacitance (C_{ox}) per unit of channel length is ~ 0.90 fF/µm for (a) and ~0.07 fF/µm for (d). (c, f) Simulated C_{ox} as a function of diameter per unit of the channel length for both structures, accordingly.

The similar simulation was also carried out on the top-gated and back-gated NWFET structures as shown in Figure S5 (a, d). The corresponding simulated gate oxide capacitance (C_{ox}) of the 60nm-diameter NW for per unit of channel length is ~ 1.12 fF/µm for the top-gated structure and ~0.07 fF/µm for the back-gated structure, and the C_{ox} for wrap-gated structure is 1.49 fF/µm.

Table S1. Current on-off ratio (I_{ON}/I_{OFF}), sub-threshold slope (*SS*), peak transconductance and peak field-effect electron mobility of wrap-gated InGaAs NWFETs.

	I_{ON}/I_{OFF}	**SS (mV/dec)**	**Peak Transconductance (μS)**	**Peak Mobility ($cm^2/(Vs)$)**
Device WG1	500000	80	2.52	1600
Device WG2	2100000	120	2.63	2900
Device WG3	25000	300	0.95	540
Device WG4	40000	225	0.47	460
Device WG5	25000	170	0.58	600
Device WG6	1000	170	2.00	1500
Device WG7	10000	250	1.85	1400
Device WG8	40000	350	1.68	1200
Average	340000	200	1.59	1300

Table S2. Current on-off ratio (I_{ON}/I_{OFF}), sub-threshold slope (*SS*), peak transconductance and peak field-effect electron mobility of back-gated InGaAs NWFETs (without any surface passivation).

	I_{ON}/I_{OFF}	SS (mV/dec)	Peak Transconductance (μS)	Peak Mobility ($cm^2/(Vs)$)
Device BG1	20000	1300	0.07	1100
Device BG2	48000	1100	0.17	760
Device BG3	9000	750	0.29	1200
Device BG4	98000	730	0.14	1600
Device BG5	200000	890	0.22	1100
Device BG6	2000	1300	0.14	810
Device BG7	30000	1060	0.17	700
Device BG8	26000	950	0.21	1100
Average	54000	1000	0.18	1050
